# *Campylobacter jejuni*-Associated Hemophagocytic Lymphohistiocytosis and Guillain-Barre Syndrome: A Case Report

**DOI:** 10.3389/fmed.2022.895923

**Published:** 2022-07-07

**Authors:** Fang-e Shi, Mei-fang Chen, Yong-jie Li, Gui-ying Dong, Ji-hong Zhu

**Affiliations:** ^1^Department of Emergency, Peking University People's Hospital, Beijing, China; ^2^Department of Infectious Diseases, Peking University People's Hospital, Beijing, China; ^3^Department of Neurology, Peking University People's Hospital, Beijing, China

**Keywords:** *Campylobacter jejuni*, hemophagocytic lymphohistiocytosis, Guillain-Barre Syndrome, peripheral nerve injury, ophthalmoplegia, facial paralysis

## Abstract

*Campylobacter jejuni* (*C. jejuni*), a Gram-negative bacterium, belongs to microaerobic bacteria. We reported a 21-year-old male patient diagnosed with hemophagocytic lymphohistiocytosis (HLH) due to *C. jejuni* infection, who presented with multiple clinical manifestations of peripheral nerve injury, such as ophthalmoplegia, facial paralysis, and urinary retention during the treatment. Electromyography showed neurogenic injury and the final diagnosis was Guillain-Barre Syndrome (GBS). After treatment of dexamethasone combined with immunoglobulin, the patient was discharged from the hospital with partial recovery of neurological symptoms.

## Introduction

*Campylobacter jejuni* (*C. jejuni*), a Gram-negative bacterium, belongs to microaerobic bacteria. We reported a 21-year-old male patient diagnosed with hemophagocytic lymphohistiocytosis (HLH) due to *C. jejuni* infection, who presented with multiple clinical manifestations of peripheral nerve injury, such as ophthalmoplegia, facial paralysis, and urinary retention during the treatment. Electromyography showed neurogenic injury and the final diagnosis was Guillain-Barre Syndrome (GBS). After treatment of dexamethasone combined with immunoglobulin, the patient was discharged from the hospital with partial recovery of neurological symptoms.

## Case Report

The patient, a 21-year-old Uygur male, had a history of fever and diarrhea for 8 days, with the highest body temperature of about 38.6°C, accompanied by chills, and sparse watery stool several times, without abdominal pain. Due to the intermittently increased temperature after taking non-steroidal anti-inflammatory drugs (NSAIDs), the patient was treated in the fever clinic but got a poor treatment effect, therefore he was admitted to the emergency department on October 10, 2021.

Physical examination on admission showed a body temperature of 38°C, pulse of 102 times/min, respiration of 23 times/min, and blood pressure of 125/72 mmHg, with clear consciousness, palpable spleen under the ribs. No other significant abnormalities were found in other physical examinations.

**Figure 1:**
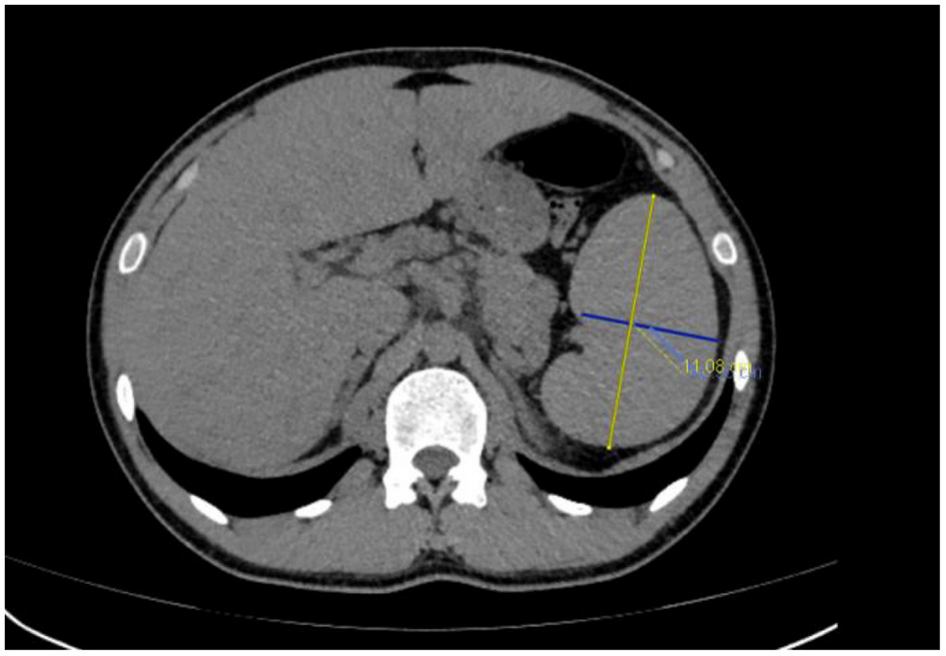
Abdominal and computed tomography (CT) revealed splenomegaly.

Emergency laboratory analysis in the emergency department indicated the following: white blood cell count decreased from normal range to 1.56 × 10^9^/L (reference value, RV 3.5–9.5 × 10^9^/L), hemoglobin was 166 g/L (RV 130–175 g/L), platelet decreased from normal range to 74 × 10^9^/L (RV 125–350 × 10^9^/L), aspartate aminotransferase was 150u/L (RV 15–40 u/L), lactate dehydrogenase was 1249 u/L (RV 109–245 u/L), triglyceride was 2.29 mmol/L (RV 0.45–1.7 mmol/L), sodium was 124.2 mmol/L (RV 137–147 mmol/L), fibrinogen decreased from normal range to 1.27 g/L (RV 2–4 g/L), sCD25 was 25,398 pg/mL (RV < 6,400pg/mL), ferritin was 3671u g/L (RV 30-400ng/mL), natural killer cell activity was 18.48% (RV ≥ 15.11%). G-test (serum (1,3)-β-D-glucan test) was positive, GM-test (galactomannan test) was negative, and blood culture, procalcitonin, c-reactive protein, erythrocyte sedimentation rate and T-SPOT.TB test were all negative. *Mycoplasma pneumoniae* antibody IgM (Passive Particle Agglutination, FUJIREBIO) was 1:80 positive (RV < 1:20), *Legionella pneumophila* antibody IgM (ELISA, Euroimmun) was 0.82 positive (RV < 0.8). The detection of Cytomegalovirus (CMV), Epstein Barr virus (EBV), Adenovirus and Coxsackievirus were all negative (real-time PCR). The oncological and immunological indexes were normal. Plain CT scan of the thoracoabdominal basin showed mild fatty liver and splenomegaly (Figure 1). There are many small lymph nodes in the hepatogastric space and retroperitoneal space.

Referring to HLH-2004 diagnostic criteria, the patient met the following conditions: ① Temperature > 38.5°C for more than 7 days; ② Hemocytopenia (WBC <10 × 10^9^/L, PLT <100 × 10^9^/L, hemoglobin 166 g/L); ③Splenomegaly; ④Low fibrinogen (FIB <1.5 g/L) and high triglycerides (2.29 mmol/L), although not meeting the diagnostic criteria (3 mmol/L); ⑤Ferritin > 500u g/L;⑥sCD25 > 6,400 pg/mL. Considering the definitive diagnosis of HLH, the patient was treated with 20 g immunoglobulin QD (8 days) and 0.5 g imipenem cilastatin sodium Q8H, and his leukocytes, platelets, and fibrinogen gradually returned to normal levels. However, on day 14 after his onset, the patient developed a series of symptoms of peripheral nerve involvement, as shown in Table 1.

**Table 1:** Disease progression timeline.

**Day**	**Symptoms and signs**	**Affected nerve**
Day 14	Headache, binocular diplopia, dilated righ mydriasis and weakened light reflex.	Oculomotor nerve
Day 15	Right eyelid ptosis	
Day 16	Right facial paralysis	Facial nerve
Day 17	Urinary retention	Autonomic nerve
Day 18	Continuous non-defecation	Autonomic nerve
Day 19	Dilated left mydriasis, weakened light reflection	Oculomotor nerve
	Mastication difficulty, corneal reflex retardation, slightly weakened bilateral biceps brachii and triceps brachii reflex, basically disappeared lower limb achilles tendon reflex and knee reflex, negative Babinski sign	Trigeminal nerve Spinal nerve
Day 27	Left facial paralysis	Facial nerve

Lumbar puncture was performed on the 16th day. Cerebrospinal fluid showed a low pressure and turbid appearance, containing 150 white cells/μL (90% lymphocytes, 10% neutrophils), protein 1.09 g/L, glucose 2.37 mmol/L, and chlorine 100.6 mmol/L; Bacteria, fungi, *cryptococcus*, and *Mycobacterium tuberculosis* were all negative; Rubella virus, CMV and EBV IgM antibody and DNA were later reported as negative. CSF and serum albumin data: CSF and serum albumin was 3.27 and 37 g/L, respectively (October 19); while 1.09 and 32.6 g/L, respectively (October 22). The albumin quotient (albumin in CSF/albumin in serum) indicated that an increased permeability of the blood-brain. No abnormality was found in CSF next-generation sequencing technology (NGS) implemented by Biotechnology Co., Ltd. Briefly, they optimized the internal index adapter and real-time analysis pipeline, established the optimal threshold for pathogen identification, and performed rapid metagenomic-NGS analysis (1). Peripheral neuropathy, demyelination, paraneoplastic tumor, autoimmune brain-related antibodies, serum antibodies IgM and IgG of *C. jejuni* were positive by enzyme-linked immunosorbent assay (ELISA), performed by Dr. Hao from the first hospital of Peking University (2), The values of IgM and IgG were greater than the negative control values (the cut-off values were 0.08 and 0.16 respectively) even after 1:160 dilution. Given the clinical presentation and CSF findings, based on intravenous dexamethasone of 20mg QD, the treatment regimen was adjusted to meropenem 1000 mg Q8H combined with acyclovir 60 mg Q8H and Mecobalamin 500 ug QD. On the 18th day, the patient's body temperature dropped to normal. On the 19th day, the lumbar puncture was performed again, and the cerebrospinal fluid became clear. CSF laboratory examination showed white blood cell count of 91/μL (92% single nucleus, 8% multiple nuclei), protein level of 3.27 g/L, normal glucose and chlorine, as well as negative NGS. Electromyography (Table 2) and cranial MRI were generally normal.

**Table 2:** Electromyography.

**Motor nerve Conduction Velocity (MCV)**						
**Nerve and Site**	**Latency**	**Amplitude**	**Segment**	**Latency Difference**	**Distance**	**Conduction Velocity**
**Peroneal.L**						
Ankle	5.5 ms	3.0 mV	Ankle-Fibula (head)	8.8 ms	350 mm	40 m/s↓
Fibula (head)	14.3 ms	2.7 mV		ms	mm	m/s
**Tibia.L**						
Ankle	4.3 ms	13.1 mV	Ankle-Popliteal fossa	9.8 ms	400 mm	41 m/s
Popliteal fossa	14.1 ms	9.6 mV		ms	mm	m/s
**Tibia.R**						
Ankle	4.6 ms	14.8 mV	Ankle-Popliteal fossa	1.7 ms	435 mm	41 m/s
Popliteal fossa	15.3 ms	11.4 mV		ms	mm	m/s
**Peroneal.R**						
Ankle	5.8 ms	0.9 mV↓78%	Ankle-Fibula (head)	8.5 ms	345 mm	41 m/s
Fibula (head)	14.3 ms	0.8 mV↓		ms	mm	m/s
**Median.L**						
Wrist	3.8 ms	10.1 mV	Wrist-Elbow	4.1 ms	235 mm	57 m/s
Elbow	7.9 ms	9.0 mV		ms	mm	m/s
**Ulnar.L**						
Wrist	2.9 ms	10.8 mV	Wrist-Below elbow	3.8 ms	230 mm	61 m/s
Below elbow	6.7 ms	9.7 mV		ms	mm	m/s
**F-wave**						
**Nerve**	**M-Latency**	**F-Latency**	**F-frequency**			
**Tibia.L**	4.1	56.6↑	100.0			
**Tibia.R**	5.3	58.5↑	100.0			
**Median.L**	4.2	28.7	100.0			
**Sensory nerve Conduction Velocity (SCV)**						
**Nerve and Site**	**Latency**	**Amplitude**	**Segment**	**Latency Difference**	**Distance**	**Conduction Velocity**
**Peroneal.L**						
Lower leg	2.8 ms	8μV	Ankle-Lower leg	2.8 ms	120 mm	43 m/s
**Sural.L**						
Lower leg	4.4 ms	17μV	Ankle-Lower leg	4.4 ms	180 mm	41 m/s
**Sural.R**						
Lower leg	4.2 ms	17μV	Ankle-Lower leg	3.7 ms	170 mm	40 m/s↓
**Peroneal.R**						
Lower leg	3.7 ms	6μV	Ankle-Lower leg	3.7 ms	140 mm	38 m/s↓24%
**Median.L**						
Wrist	2.7 ms	41μV	Digit II (finger)-Wrist	2.7 ms	145 mm	54 m/s
**Median.L**						
Wrist	2.4 ms	44μV	Digit III (middle finger)-Wrist	2.4 ms	145 mm	59 m/s
**Ulnar.L**						
Wrist	2.3 ms	41μV	Digit V (little finger)-Wrist	2.3 ms	125 mm	54 m/s
**Radial.L**						
Forearm	2.3 ms	19μV	Anatomical snuff box-Forearm	2.3 ms	135 mm	59 m/s
**H-wave**						
**Nerve**	**Latency**	**Amplitude (max)**				
**Tibia.L**						
M-wave	4.0 ms	2.6 mV				
H-wave	46.6 ms, poor form of wave, incubation period↑, frequency↓	0.4 mV				
**Tibia.R**						
M-wave	5.3 ms	16.2 mV				
H-wave	Not elicited	Not elicited				

Considering the peripheral nerve injury and cerebrospinal fluid protein cell separation, GBS secondary to *C. jejuni* infection was specifically classified as Miller Fisher syndrome (MFS) and Bickerstaff brainstem encephalitis. On the 23rd day of onset, the diameter of the right pupil gradually recovered to 4 mm the pupillary light reflex, and the closing function of the right upper eyelid gradually recovered. On the 26th day, dexamethasone was reduced to 10 mg QD. On the 27th day, the left pupil diameter recovered to about 4 mm, and the pupillary light reflex recovered. On the 28th day, the patient defecated autonomously. On the 41st day, spontaneous urination recovered, and the patient was hospitalized for 42 days. Before discharge, the patient had left bilateral facial paralysis and mild diplopia in both eyes. One month after discharge, the patient was followed up, leaving slight facial paralysis, and other symptoms returned to normal.

## Discussion

*C. jejuni*, is a kind of Gram-negative bacteria, with which humans are easily infected. The incidence rate is high in summer and autumn, and the number of infected men is about 1.2–1.5 times that of women (3). *C. jejuni*, is the leading cause of bacterial diarrhea with an incubation period of about 2–7 days. The clinical manifestations are characterized by fever, abdominal pain, and red and white blood cells in feces. Parenteral infection is common in patients aged 40–70 or with hypo-immunity. Blood-borne infection can cause complications such as bacteremia and meningitis.

GBS is a group of acute autoimmune-mediated peripheral neuropathys, generally with a history of precursor infection in two-thirds of patients, and its common pathogens include *C. jejuni*, CMV, EBV, *M. pneumonia*, etc, of which *C. jejuni* is the most common (4). The patient was positive for *M. pneumonia* IgM antibody and weakly positive for *L. pneumophila* IgM antibody, we consider these to be false-positive results because the patient's body temperature dropped to normal without targeted treatment (5, 6). At the same time, GBS is also one of the serious complications caused by *C. jejuni* infection (7). Most of the clinical symptoms of GBS peak in about 2 weeks. The typical clinical manifestation is paresthesia of the distal limbs, accompanied by symmetrical weakness of the lower limbs, and then involving the muscles innervated by the upper limbs and cranial nerves. Autonomic dysfunction is also a common symptom, including abnormal pupil, intestinal, or bladder function. Protein-cell separation can be seen in cerebrospinal fluid. Nerve conduction velocity and electromyography contribute to GBS diagnosis and primary myelin injury determination. The infection rate of *C. jejuni* in GBS patients with diarrhea as a precursor is as high as 85%. In 50% of patients with C. *jejuni* enteritis, the bacteria cannot be isolated by fecal examination 2 weeks after diarrhea stops (7).

Acute inflammatory demyelinating polyneuropathy (AIDP) and acute motor axonal neuropathy (AMAN) are the two most common subtypes of GBS. The less common subtypes include acute motor-sensory axonal neuropathy (AMSAN) and Miller Fisher syndrome (MFS) (7). Asia has a high incidence rate of MFS, and the proportion of GBS patients is approximately 15–25%. The main clinical manifestations are ophthalmoplegia, ataxia, weakening, or disappearance of tendon reflex. The initial symptoms are diplopia and limb weakness. Neurogenic injury is common on EMG, with decreased nerve conduction velocity, and prolonged or absent F wave (8). The etiology and pathogenesis of MFS remain unclear. They are mainly related to the autoimmune response after infection. It is currently believed that antibodies generated after *C. jejuni* infection directly attack the presynaptic membrane and synaptic space (9, 10), resulting in impaired integrity of peripheral nerves and loss of nerve conduction-related functions. Recent studies have shown that anti-GQ1b, anti-GD1b, and anti-GT1a antibodies are related to the pathogenesis of MFS, among which anti-GQ1b is the most specific, mainly deposited in the paraganglionic area of oculomotor nerve, trochlear nerve, and abductor nerve, with the most abundant content in oculomotor nerve. After the occurence of clinical symptoms, the antibody titer increases rapidly and then decreases when the symptoms turn negative. Generally, the serum anti-GQ1b antibody turns negative one month after onset (11). Combined with the neurological manifestations, as well as the increased lymphocyte number and protein content in CSF, the diagnosis of the patient is closer to MFS and Bickerstaff brainstem encephalitis.The IgM and IgG antibodies of C. *jejuni* were positive in the blood samples while the ganglioside antibodies were negative in the cerebrospinal fluid. There are two possibilities to explain this false-negative phenomenon. Firstly, some studies have found that about 10–30% of MFS cases are negative in the routine detection of GQ1b antibodies. Due to the fact that GQ1 antibodies are calcium dependent, they may fail to bind the detection ligand in the absence of sufficient Ca^2+^. Secondly, rapid antibody depletion can also result in a negative test result (12, 13).

HLH, a reactive disease caused by infection, tumor, immune-related diseases, and other causes that activate the immune system, is mainly characterized by persistent fever, hepatosplenomegaly, pancytopenia, and hemophagocytosis in the bone marrow and lymphoid tissue (14, 15). HLH is divided into primary HLH and secondary HLH. Primary HLH is an autosomal or sex chromosome recessive disease, and secondary HLH is related to various underlying diseases.

Currently, there is no report of HLH caused by *C. jejuni*. A case report of HLH complicated with GBS caused by EB virus infection was found in the search database (16). *C. jejuni* has not been reported to simultaneously cause both HLH and GBS. After excluding other diagnostic possibilities, we considered that HLH was caused by *C. jejuni* infection, and then GBS appeared as a consequence of an autoimmune response.

GBS is generally considered to be elicited by diverse ganglioside antibodies or multiple antibody complexes generated by varying serotypes and genotypes of *C. jejuni*. It's been reported that the lipo-oligosaccharide (LOS) motifs of *C. jejuni* isolated from GBS patients resemble peripheral nerve gangliosides (GM1, GD1a, GQ1b, etc.). The key antigenic molecule is presumably the sialylated oligosaccharide motif on the LOS of *C. jejuni*, which contains the heptosyl residue peculiar to LOS and the oligosaccharide component simulating gangliosides. Molecular mimicry has significant effect on the production of the antibodies. A subtle degree of structural differences of key antigenic molecules in the pathogen may trigger a breakdown of tolerance and lead to autoimmunity (17). The combination of antigen and antibody, meanwhile, will elicit inflammatory cell infiltration and complement fixation, which would cause immune attacks on specific sites of peripheral and central nerves with similar antigenicity, thereby resulting in nerve injury. The key motif is also the ligand of Siglec-1 (CD169) needed for phagocytosis by antigen presenting cells (APC). Siglec-1 is a type1 transmembrane protein and belongs to the Sialic acid binding Ig-like lectin family. It plays a critical role in the maturation of colonic T cells and in the elicitation of autoantibodies (18). Furthermore, a recent study has found that GBS patients exhibit defective signaling of Siglec-10, an inhibitory receptor expressed by B cells, which may give rise to aberrant B cell responses against gangliosides or LOS. This indicates that there probably exists a regulatory mechanism that will restrict the risk of autoimmunity to self-glycolipids, and this mechanism may be undermined in patients with GBS (17).

In conclusion, given the irreversible damage of GBS, early identification and timely treatment of central nervous system diseases caused by GBS can prevent irreversible injury to the central nervous system, playing a vital role in the improvement of the long-term prognosis.

## Data Availability Statement

The original contributions presented in the study are included in the article/supplementary material, further inquiries can be directed to the corresponding author/s.

## Ethics Statement

Written informed consent was obtained from the individual(s) for the publication of any potentially identifiable images or data included in this article.

## Author Contributions

All authors listed have made a substantial, direct, and intellectual contribution to the work and approved it for publication.

## Conflict of Interest

The authors declare that the research was conducted in the absence of any commercial or financial relationships that could be construed as a potential conflict of interest.

## Publisher's Note

All claims expressed in this article are solely those of the authors and do not necessarily represent those of their affiliated organizations, or those of the publisher, the editors and the reviewers. Any product that may be evaluated in this article, or claim that may be made by its manufacturer, is not guaranteed or endorsed by the publisher.
